# Fucoidan inhibition of lung cancer *in vivo* and *in vitro*: role of the Smurf2-dependent ubiquitin proteasome pathway in TGFβ receptor degradation

**DOI:** 10.18632/oncotarget.2317

**Published:** 2014-08-06

**Authors:** Hsien-Yeh Hsu, Tung-Yi Lin, Yu-Chung Wu, Shu-Ming Tsao, Pai-An Hwang, Yu-Wei Shih, Jason Hsu

**Affiliations:** ^1^ Department of Biotechnology and Laboratory Science in Medicine, National Yang-Ming University, Taipei, Taiwan; ^2^ The Genomics Research Center, Academia Sinica, Taipei, Taiwan; ^3^ Program in Molecular Medicine, National Yang-Ming University and Academia Sinica, Taipei, Taiwan; ^4^ Division of Thoracic Surgery, Department of Surgery, Taipei Veterans General Hospital, Taipei, Taiwan; ^5^ School of Medicine, National Yang-Ming University, Taipei, Taiwan; ^6^ Seafood Technology Division, Fisheries Research Institute, Council of Agriculture, Keelung, Taiwan; ^7^ Fordham University, New York, NY, USA

**Keywords:** Fucoidan, lung cancer, transforming growth factor β receptors (TGFRs), Smad ubiquitination regulatory factor 2 (Smurf2), ubiquitination proteasome pathway (UPP)

## Abstract

Fucoidan, a polysaccharide extracted from brown seaweeds, reduces tumor cell proliferation. In this study, we demonstrate that fucoidan reduces tumor size in LLC1-xenograft male C57BL/6 mice. Moreover, we found that LLC1-bearing mice continuously fed fucoidan showed greater antitumor activity than mice with discontinuous feeding. Fucoidan inhibited the *in vitro* growth of lung cancer cells. Transforming growth factor β (TGFβ) receptors (TGFRs) play important roles in the regulation of proliferation and progression, and high TGFRI expression in lung cancer specimens is associated with a worse prognosis. Herein, using lung cancer cells, we found that fucoidan effectively reduces TGFRI and TGFRII protein levels *in vivo* and *in vitro*. Moreover, fucoidan reduces TGFR downstream signaling events, including those in Smad2/3 and non-Smad pathways: Akt, Erk1/2, and FAK phosphorylation. Furthermore, fucoidan suppresses lung cancer cell mobility upon TGFβ stimulation. To elucidate how fucoidan decreases TGFR proteins in lung cancer cells, we found that fucoidan enhances the ubiquitination proteasome pathway (UPP)-mediated degradation of TGFRs in A549 and CL1-5 cells. Mechanistically, fucoidan promotes Smurf2 and Smad7 to conjugate TGFRs, resulting in TGF degradation; however, Smurf2-shRNA abolishes fucoidan-enhanced UPP-mediated TGFR degradation. Our study is the first to identify a novel mechanism for the antitumor activity of fucoidan, namely decreasing tumor growth by modulating the TGFR/Smad7/Smurf2-dependent axis, leading to TGFR protein degradation and inhibition of lung cancer cell progression *in vitro* and *in vivo.* Our current findings indicate that fucoidan is a potential therapeutic agent or dietary supplementation for lung cancer, acting via the Smurf2-dependent ubiquitin degradation of TGFβ receptors.

## INTRODUCTION

Fucoidan is an aggregate name for algal fucose-enriched sulfated polysaccharides extracted from the extracellular matrix of brown seaweeds. The structure of fucoidan principally consists of an α-1,3-backbone or a repeat unit comprising disaccharides containing α-1,3-linked fucose and α-1,4-linked fucose, i.e., α (1→3)- and α (1→4)-bonded L-fucopyranose residues with additional branches attached at the C2 positions. The L-fucopyranose residues may be substituted with a sulfate group (SO_3_^−^) on the C-2 or C-4 (rarely on C-3) position, with single L-fucosyl residues and/or with short side chains of fucoside (fuco-oligosaccharide) (Supplemental data, Scheme A) [1, 2]. The biological activities of fucoidan have been examined and reported elsewhere [2, 3]; in particular, the anti-inflammatory, anti-proliferative, and anti-cancer/anti-tumor activities of fucoidan have recently drawn considerable attention. We recently reported that fucoidan inhibits breast cancer cell growth *in vitro* and *in vivo* via the involvement of ubiquitin proteasome pathway (UPP)-mediated transforming growth factor β receptor (TGFR) degradation [4]. Fucoidan also induces apoptosis by the activation of caspase 3 and downregulation of Erk-mediated pathways [5] as well as by the activation of caspases 9 and 8, which inhibit the growth of A549 (human lung adenocarcinoma) cells [6] and MCF-7 (human breast cancer) cells [7], respectively. In addition, fucoidan inhibits invasion and angiogenesis by human fibrosarcoma cells via repression of the activities of matrix metalloproteinases 2 and 9 [8].

Lung cancer is among the leading causes of cancer-related deaths (mortality) in humans worldwide, accounting for more than 1.3 million deaths each year [9, 10]. Lung cancer has a higher mortality rate due to its ability to metastasize early from the lungs to distant organs. In general, lung cancers can be broadly divided into two major forms: non-small cell lung cancer (NSCLC) and small-cell lung cancer (SCLC). Lung adenocarcinoma, a subtype of NSCLC, represents the most common histological type of lung cancer [11]. The treatment of lung cancer is generally performed using surgery, chemotherapy, radiation therapy, and target therapy [9]. Although physicians have been dedicated to improving the treatment and management of lung cancer, the survival rate of lung cancer remains low. Most patients with advanced NSCLC will have had their disease metastasize, and the five-year survival rate is less than 15% [12].

Transforming growth factor β1 (TGFβ1) plays a dual role in cancer biology, in both tumor suppression and tumor promotion [13]. The over-expression of TGFβ1 promotes tumor growth and aggressive pulmonary metastasis during the late stages of lung carcinogenesis [13-15]. High TGFβ1 expression represents an important prognostic parameter after surgical resection for patients with NSCLC [16]; indeed, TGFβ1 plays critical and essential roles in the tumor progression and metastasis of lung cancers [17, 18]. In addition, TGFβ is described as a tumor promoter, with the ability to induce the epithelial to mesenchymal transition (EMT) [19, 20]. The canonical signaling events induced by TGFβ1 begin by the binding of ligands to the TGFβ type II receptor (TGFRII), which then recruits the TGFβ type I receptor (TGFRI) to form a complex in which TGFRI is activated [21]. Subsequently, the activated TGFRI directly phosphorylates Smads, namely Smad2 and Smad3, which associate with Smad4 and then translocate into the nucleus, regulating the expression of target genes [22, 23]. In the TGFβ1 non-canonical pathway (the so-called non-Smad pathway), the activated TGFR complex transmits a signal through other factors, such as TAK1, PI3K-AKT, ERK, focal adhesion kinase (FAK), and p38, [24-26], which also mediate tumor progression, mobility, and metastasis in human lung adenocarcinoma [27].

TGFR degradation signaling has been reported to be regulated by ubiquitin-dependent proteasomal pathways (UPPs) [4, 28]. In general, ubiquitination controls the turnover of short-lived proteins in a cell. The ubiquitination process involves the activation of three specific enzymes, including ubiquitin-activation enzyme (E1), ubiquitin-conjugation enzyme (E2), and ubiquitin ligase enzyme (E3) [29], which regulate ubiquitin molecules to attach to specific target proteins. Subsequently, these polyubiquitinated target proteins are disrupted and degraded by the 26S proteasome complex. The Smad ubiquitination regulatory factor 2 (Smurf2), a specific C2-WWHECT-domain E3 ligase, participates in modulating TGFβ-mediated signaling by targeting TGFR and Smad2. However, Smad7 is one of the key negative regulators of the TGFβ signaling pathway because Smad7 acts as an adaptor protein to help Smurf2 conjugate to TGFR, an event that is followed by ubiquitination processes [30, 31].

In our current study, we demonstrate that fucoidan inhibits the viability of human NSCLC cells and mouse lung cancer cells, reduces lung tumorigenesis (tumor volume and weight), and inhibits TGFRI/II protein expression in LLC1-xenograft mice orally fed with fucoidan. Our novel findings suggest that fucoidan enhances the Smurf2-mediated ubiquitination of TGFR and consequently TGFR degradation. In parallel, we demonstrate that fucoidan inhibits TGFβ/TGFR downstream Smad and non-Smad pathways (FAK, Akt and Erk) and suppresses cell mobility. Our findings suggest that fucoidan is a promising therapeutic agent for the prevention of lung tumorigenesis.

## RESULTS

### Fucoidan suppresses tumorigenesis and reduces transforming growth factor β (TGFβ) receptor (TGFR) protein levels in LLC1 cell-xenograft male C57BL6 mice *in vivo*

**Figure 1:**
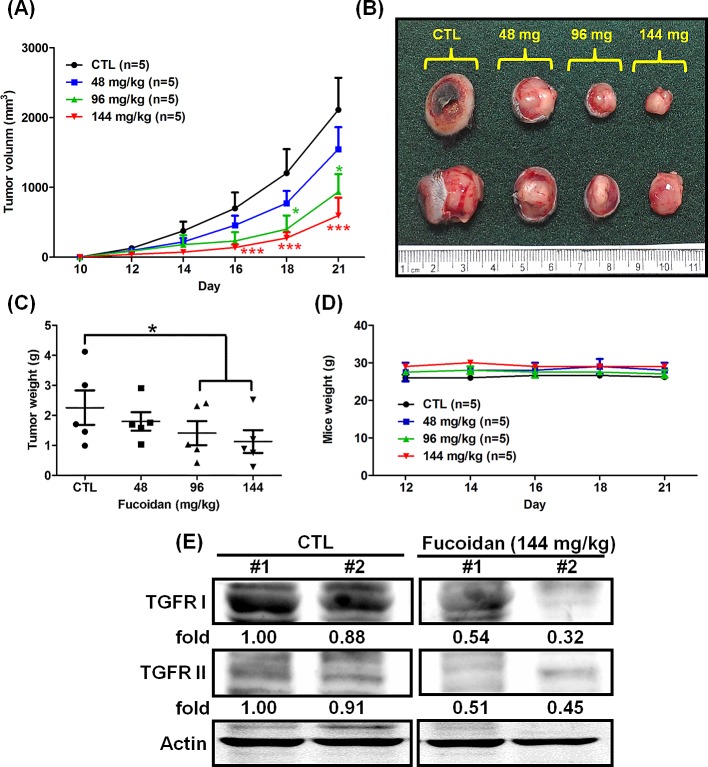
Fucoidan inhibits tumorigenesis in LLC1 cell-xenograft male C57BL6 mice LLC1 cells (2 × 10^5^) were inoculated into the hypodermic dorsum of male C57BL6 mice. Beginning on the seventh day, the mice were orally fed with ddH_2_O (control) and 48/96/144 mg of fucoidan per kg for 21 days at 7 oral feedings/week/mouse. (A) The tumor volume was measured, and (B) tumor samples from the mice were collected at the end of treatment. (C) The tumor weight and (D) mouse weight were also measured. (E) The TGFR protein levels of the tumor lesions were assessed. Western blot analysis was performed to determine the TGFR protein levels. Two of five individual experiments are presented. Each bar represents the mean ± SEM. Significant differences are shown (*P < 0.05 and ***P < 0.005, compared with the control group).

Our previous finding demonstrated that fucoidan suppresses the tumorigenesis and metastasis of breast cancer [4]. Herein, we further examined the effect of orally fed fucoidan on mice with Lewis lung carcinoma (LLC1)-xenograft *in vivo*. LLC1 cells were inoculated into the hypodermic dorsum of male C57BL6 mice, and the tumor growth rate was assessed over 21 days. We found that mice fed with fucoidan showed a marked dose-dependent reduction in tumor volume (Figs. 1A and 1B) and tumor weight (Fig. 1C); in contrast, less of a difference was observed in the mouse body weight between the fucoidan-fed mice and ddH_2_O control groups (Fig. 1D). Using western blot analyses to verify the TGFR expression of the tumor lesions, we found that there was less expression of TGFRs (TGFRI and II) in fucoidan-fed LLC1-bearing mice than in the control group (Fig. 1E), indicating that fucoidan down-regulates TGFRI and TGFRII protein expression in tumor lesions *in vivo*. Together, these results show that fucoidan suppresses tumorigenesis and reduces TGFR protein expression in an LLC1-bearing mouse model *in vivo*. Alternatively, to examine the degree of liver injury in mice fed with fucoidan, the AST and ALT levels in mice were analyzed using tail vein blood collection at the end of fucoidan treatment. Our results indicated neither evidence of fucoidan affecting liver functions nor liver toxicity in the mouse model (Supplementary Figs. 1A and 1B).

### Greater efficacy in the reduction of tumor volume/weight and inhibition of TGFRI/II protein expression in LLC1-bearing mice orally fed continuously with fucoidan

Considering that fucoidan inhibits tumor growth in LLC1-bearing mice, we hypothesized that oral feeding with fucoidan plays a pivotal role in the manipulation of tumor growth/size in LLC1-bearing mice. To test this hypothesis, we examined the tumor volume in mice with or without oral feeding with fucoidan during the growth of tumor-bearing mice and compared the tumor volume in mice orally fed with fucoidan or ddH_2_O (CTL) during the entire testing period (Supplementary Fig. 2). Briefly, we separated the mice into three separate treatment groups with fucoidan: continuous feeding (EXP 3), discontinuous feeding (EXP 2), and day 13-start feeding (EXP 1).

**Figure 2:**
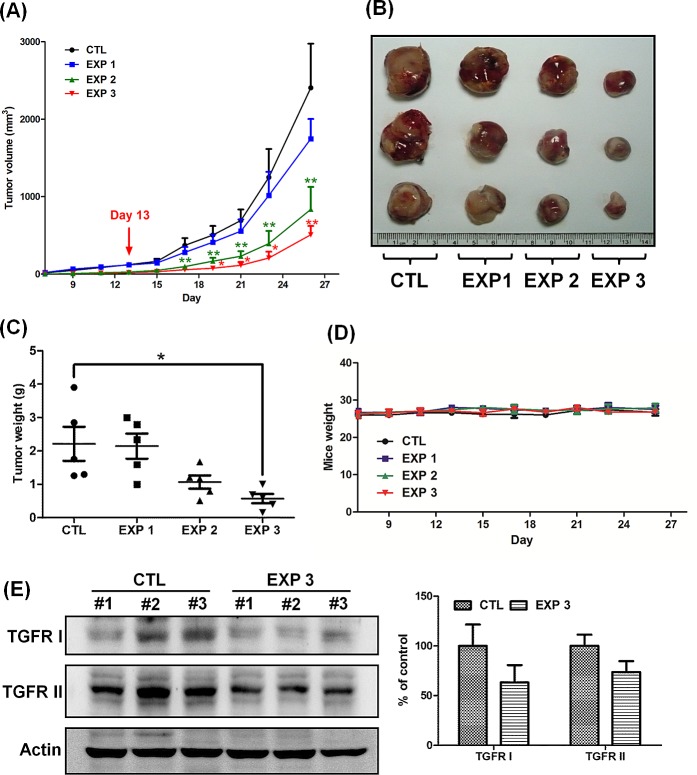
Continuous treatment of fucoidan has a greater efficacy in reducing the tumor volume and inhibits the expression of TGFRI and TGFRIL LLC1 cells (2 × 10^5^) were inoculated into the hypodermic dorsum of male C57BL6 mice; on the 7th day, the mice were divided into four groups and processed in a “control-start-stop-continuous” fashion (Supplementary Fig. 2). Briefly, the mice were separated into four groups: those orally fed only with ddH_2_O (control); those that began receiving oral feeding of fucoidan on day 13 (EXP 1); those that ceased receiving oral feeding of fucoidan on day 13 (EXP 2); and those that were continuously fed fucoidan for all 26 days (144 mg/kg) (EXP 3). The groups that were fed with fucoidan were fed 7 times/week/mouse. (A) The tumor volume was measured, and (B) tumor samples from the mice were collected. (C) The tumor weight and (D) mouse weight were also measured. At the end of treatment, (E) the TGFR proteins were analyzed from the lysates of the LLC1-xenografted tumors by western blotting. Three of five individual experiments are presented (n = 5). Each bar represents the mean ± SEM. Significant differences are shown (*P < 0.05 and **P < 0.01, compared with the control group).

After the indicated treatments, the mice on the EXP 1 protocol had significantly smaller tumor volumes and weights than those of the mice on the CTL protocol (Figs. 2A–2C). Additionally, the tumor volumes and weights of the mice on the EXP 2 protocol were significantly greater than those of the mice on the EXP 3 protocol (Figs. 2A–2C), indicating that continuous oral feeding of fucoidan has a greater efficacy in suppressing tumorigenesis, as expected. Correspondingly, the EXP1 fucoidan treatment had a lower efficacy in inhibiting tumor growth than the EXP 3 or EXP 2 treatments. Fucoidan could also attenuate tumor growth in LLC1-bearing mice in the EXP 1 group compared with the CTL group. There was no difference in body weight among the tested mice during the testing period (Fig. 2D). We also examined the expression of TGFRs in the tumor lesions and found lower expression of TGFRs in the EXP 3 group than in the CTL group (Fig. 2E), similar to the results in Fig. 1E. Taken together, these results support the notion that continuous oral feeding of fucoidan efficiently suppresses lung tumorigenesis in mice.

### Fucoidan inhibits viability and decreases TGFR-mediated Smad and non-Smad pathway activity in lung cancer cells *in vitro*

**Figure 3:**
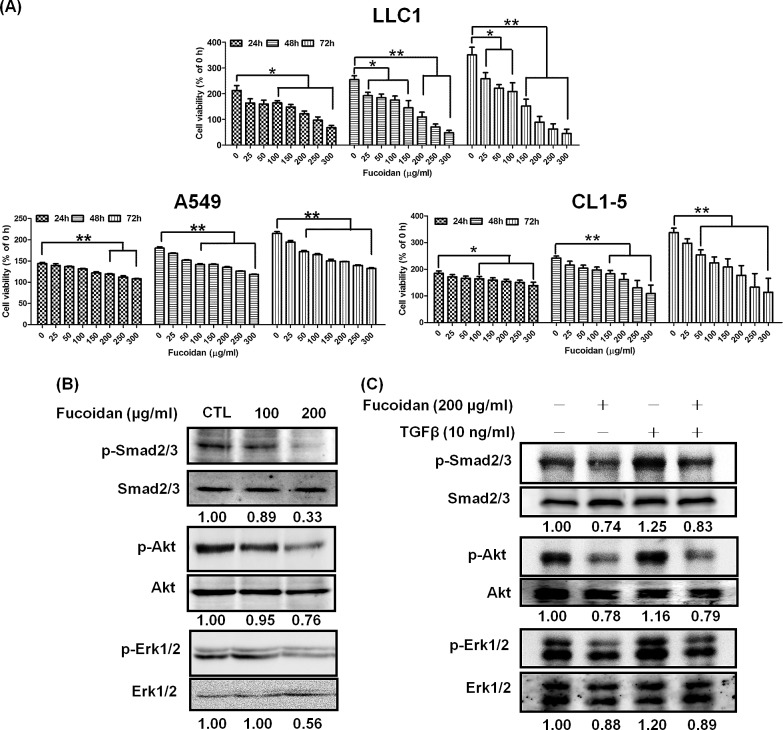
Fucoidan inhibits proliferation and decreases TGFR-mediated Smad and non-Smad pathway activity in lung cancer cells Fucoidan inhibits the proliferation of lung cancer cells (A) A549, CL1-5, and LLC1. The cells were treated with various doses of fucoidan (0–300 μg/ml) for 24, 48, and 72 h; the viability of the cells was determined using the MTT assay. Each group of fucoidan-treated samples was normalized against each untreated control. The data are representative of three separate experiments and are presented as the mean ± SD; error bars indicate SD. Significant differences are shown (*P < 0.05 and **P < 0.01, compared with the control group). (B) CL1-5 cells were treated with fucoidan (100 or 200 μg/ml) for 1 h. (C) CL1-5 cells were pre-treated with fucoidan (200 μg/ml) for 2 h and then treated with TGFβ (10 ng/ml) for 1 h. The treatments were followed by western blotting analyses of whole cell lysates to detect the expression of p-Smad2/3 (Ser423/425), p-Akt (Ser473), p-Erk1/2 (Thr202/Tyr 204). Smad2/3, Akt and Erk were used as internal controls for loading.

Because we demonstrated that fucoidan suppresses the tumorigenesis of an LLC1-xenograft mouse model *in vivo*, we next examined the effect of fucoidan on human NSCLC cells, A549 and CL1-5, and on mouse LLC1 cells *in vitro*. We used the MTT assay to examine the effect of fucoidan on the growth pattern of these lung cancer cells at 24, 48 and 72 h and found 40–60% inhibition of cell viability after 72 h of fucoidan treatment (at 100 and 200 μg/ml) in these cells compared with the control (Fig. 3A). We further examined the synergistic effect of fucoidan combined with cisplatin and found synergistic cytotoxic effects on LLC1 (Supplementary Fig. 3).

Our data showed that fucoidan results in the low expression of TGFRs in tumor lesions in LLC1-xenograft mice (Figs. 1E and 2E). Because the molecular networks of TGFR, including Smad pathway (Smad2/3) and non-Smad pathway molecules (e.g., Akt and Erk), regulate cancer cell survival and metastasis [6, 32], we examined whether fucoidan suppresses the activity of Smad and non-Smad pathways. As shown in Fig. 3B, fucoidan decreased the phosphorylation of Smad2/3, Akt and Erk in CL1-5 cells. Next, using Akt and Erk inhibitors, we found that blockade of Akt and Erk signaling abolished TGFβ1-stimulated cell viability (data not shown). Furthermore, using TGFβ1 to stimulate TGFR downstream signaling pathways, we found that TGFβ1 stimulation enhanced the phosphorylation of Smad2/3, Akt, and Erk in NSCLC; however, fucoidan still abolished the TGFβ1-induced phosphorylation of these molecules (Fig. 3C). Taken together, these data showed that fucoidan inhibition of cell viability in lung cancer cells partly involves targeting TGFR, with the consequent attenuation of the Smad2/3, Akt, and Erk signaling pathways.

### Fucoidan accelerates the degradation of TGFRI/II and reduces the stability of TGFRI/II

**Figure 4:**
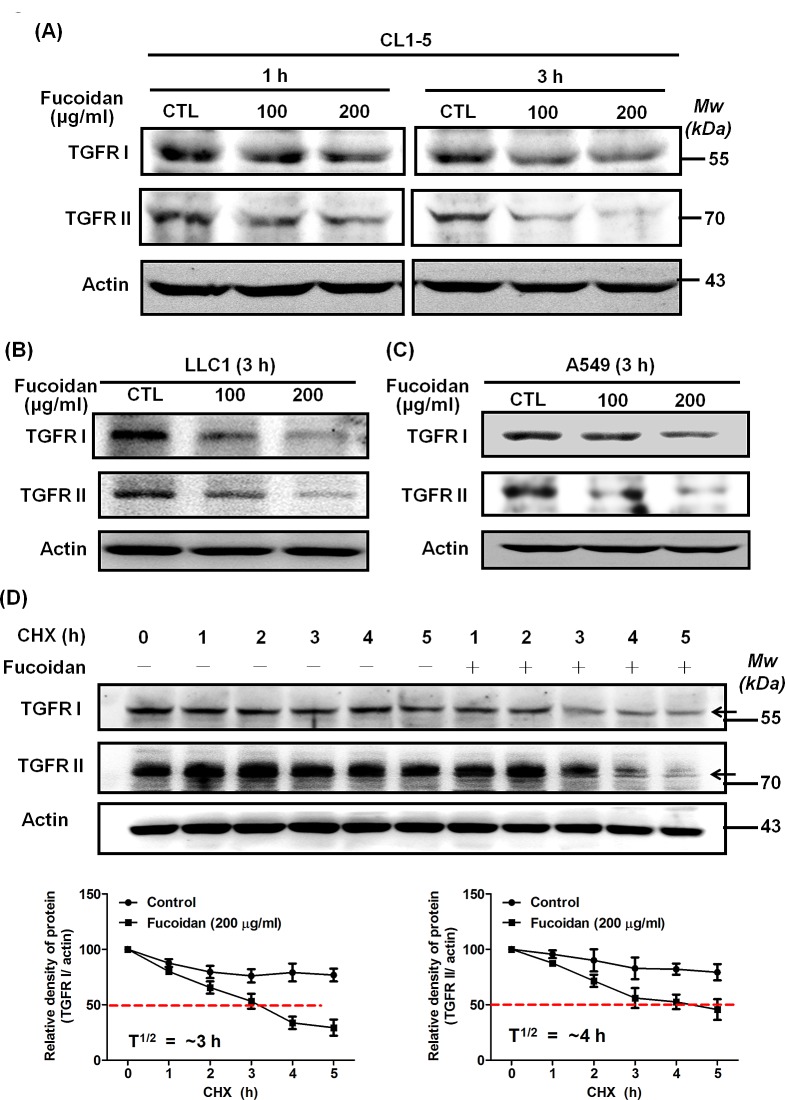
Fucoidan reduces the expression of TGFRI and TGFRII proteins by potentially accelerating protein degradation in NSCLCs Lung cancer cells CL1-5 (A), LLC1 (B), and A549 (C) were treated with fucoidan (100 and 200 μg/ml) for 1 or 3 h, respectively, followed by western blotting of whole cell lysates to detect the expression of TGFRI (V-22) and TGFRII (C-16). Actin was used as an internal control. This experiment is representative of three similar experiments. (D) Time-course studies of TGFRI and TGFRII degradation. Cycloheximide (CHX, 100 μg/ml) was added to CL1-5 cells in the presence and absence of fucoidan (200 μg/ml) for 5 h, followed by western blotting analysis. TGFRI and TGFRII bands are observed and are indicated by arrows (upper panel). Quantification of the intensities of the bands of TGFRI (left lower panel) and TGFRII (right lower panel) in the experiment are representative of three separate determinations by ImageJ (National Institute of Mental Health, Bethesda, MD, USA); the relative protein levels in each band from the cell samples were quantified by densitometry as a function of time (lower panels), with the dotted line (-----) indicating the half-life (T½) of the TGFRI and II proteins.

To investigate how fucoidan decreases TGFRs inisolated tumor lesion samples from LLC-bearing mice, we examined the effect of fucoidan on TGFRI/II protein expression in various lung cancer cells such as CL1-5, A549 and LLC1. The results of western blotting analyses showed that fucoidan rapidly reduces TGFRI and TGFRII protein expression in these cells in a dose- and time-dependent fashion compared with the levels in control cells (Figs. 4A–4C and Supplementary Figs. 4 and 5) but does not down-regulate the mRNA levels of these TGFRs upon fucoidan treatment (data not shown). In addition, we found that fucoidan down-regulates the expression of TGFRs for 24 h.

Next, using cycloheximide (CHX), an inhibitor of *de novo* protein synthesis, we further dissected the effect and function of fucoidan on the stability of the TGFRI/II proteins. Initially, we found that the half-life of TGFRs in CL1-5 cells was much longer when the cells were exposed to cycloheximide (CHX) alone (Fig. 4D). However, when the cells were co-treated with fucoidan and CHX, the degradation of TGFRI and TGFRII was significantly accelerated–i.e., the stability of TGFRs was dramatically reduced (Fig. 4D), a finding that is consistent with that in our previous reports [4]. In essence, the half-life of the TGFRI and TGFRII proteins in cells upon the co-treatment of fucoidan and CHX decreases to ~3 h and ~4 h, respectively, compared with that in cells treated with CHX alone (approximately longer than 24 h for both) (Fig. 4D). These results indicated that fucoidan has a unique function and activity in the acceleration of TGFR degradation in lung cancer cells.

### Fucoidan reduces TGFRI/II protein expression in lung cancer cells via the acceleration of ubiquitin-dependent proteasome pathway (UPP)-mediated degradation

**Figure 5:**
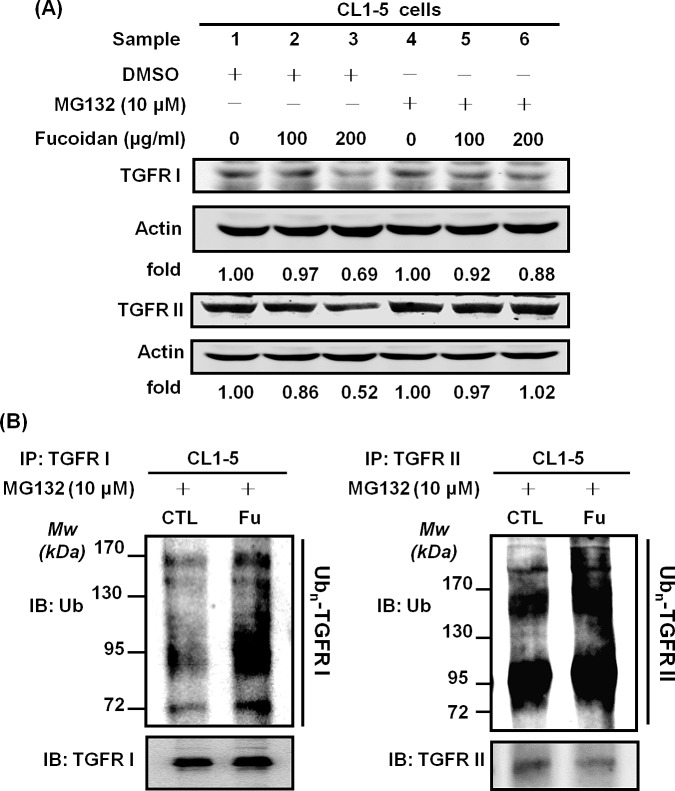
Fucoidan enhances the proteasome-mediated degradation/ubiquitination of TGFRs in NSCLCs CL1-5 cells were treated with MG132 (30 min) followed by incubation with fucoidan (100–200 μg/ml, 3 h) and then harvested for immunoblotting with (A) TGFRI and TGFRII antibodies. The detection of ubiquitin (Ub) levels was assessed by the immunoprecipitation of TGFRI and TGFRII (B) in CL1-5 cells. The cells were pre-treated with MG-132 (10 μM) for 30 min, followed by additional incubation with fucoidan (200 μg/ml) for 1 h. The procedure of TGFR immunoprecipitation was previously described in the Materials and Methods section. The results showed the elevation of poly-ubiquitin expression in the presence of fucoidan; the ubiquitin (Ub) and TGFRI/II levels were measured by western blotting analysis. This experiment is representative of three similar experiments.

One possible mechanism by which fucoidan-induced decreases in the TGFRI and TGFRII proteins might occur in NSCLC, as has been previously reported, is through ubiquitin-dependent proteasome pathway (UPP)-mediated degradation. As expected, cells incubated with fucoidan (200 μg/ml) showed 30% and 50% decreases in the protein levels of TGFRI (Fig. 5A, upper panel, samples 1 *vs* 3) and TGFRII (Fig. 5A, lower panel, samples 1 *vs* 3), respectively. We also found that the addition of MG-132, a proteasome inhibitor, to the cells could prevent the fucoidan-induced protein degradation of TGFRI (Fig. 5A, upper panel, samples 2, 3 *vs* samples 5, 6) and TGFRII (Fig. 5A, lower panel, samples 2, 3 *vs* samples 5, 6) compared with the cells treated with fucoidan alone (Fig. 5A, upper and lower panels, sample 1). These results suggest that a proteasome-dependent pathway is involved in the fucoidan-induced degradation of the TGFRI/II proteins. Moreover, when cells were pre-treated with MG-132 followed by incubation of the cells with or without fucoidan, we found no significant difference in the protein expression of TGFRI (Fig. 5A, upper panel, sample 1 *vs* 4) and TGFRII (Fig. 5A, lower panel, samples 1 *vs* 4).

Next, to further explore the mechanism of the fucoidan-induced decrease in TGFRI/II proteins, an *in vitro* ubiquitination activity assay was conducted against ubiquitin proteins to examine the role and involvement of ubiquitin (ubiquitination) in the fucoidan-mediated proteasome degradation of the TGFR proteins in NSCLC (CL1-5 and A549). The cells were pre-incubated with MG-132 and treated with fucoidan, and then the whole cell lysates were incubated with anti-TGFR antibodies. The immunoprecipitated proteins were next assessed using an anti-ubiquitin antibody. We found that the degenerated TGFRI/II proteins could be detected using an anti-ubiquitin antibody, indicating that TGFRI/II proteins were ubiquitinated during the fucoidan-induced decrease in TGFR proteins. Specifically, we demonstrated that the intensity of the smeared bands of degenerated TGFRI/II proteins in fucoidan-treated cells was stronger than that in control cells (Figs. 5B-1 and 5B-2). Similar results were found in ubiquitination activity assays of A549 cells (data not shown). Taken together, these results indicate that the smeared bands consisted of polyubiquitinated TGFRI and II proteins via the involvement of ubiquitin-proteasome pathways (UPPs) in fucoidan-enhanced TGFR degradation in NSCLC.

### Fucoidan enhances the conjugation of Smad ubiquitination regulatory factor 2 (Smurf2) and Smad7 on TGFRs, and Smurf2-shRNA (sh-Smurf2) interferes/disturbs the fucoidan-induced degradation of TGFRs

**Figure 6:**
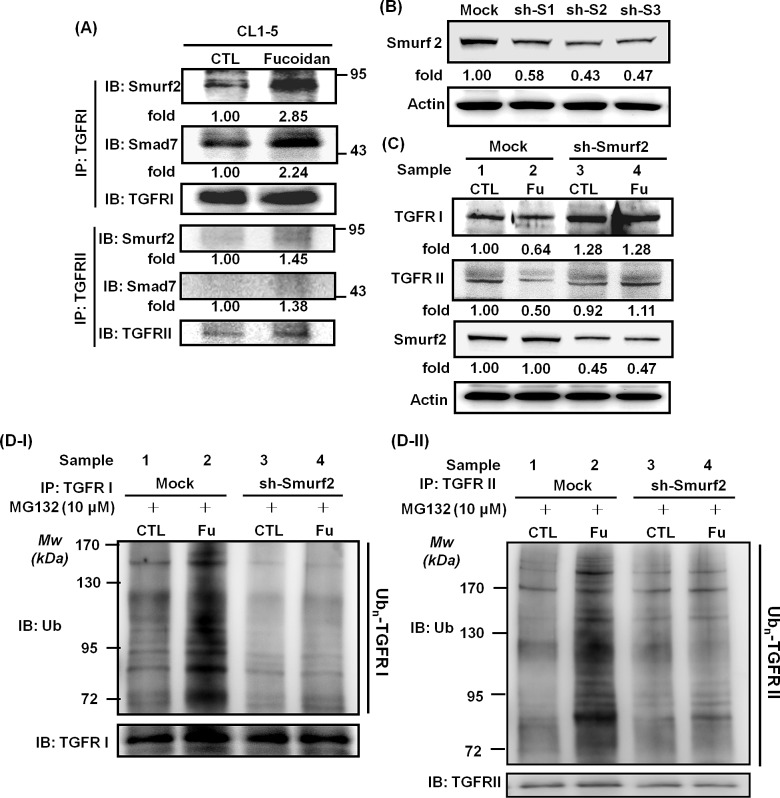
Fucoidan enhances the conjugation of Smurf2/Smad7 on TGFRs, and Smurf2-shRNA (sh-Smurf2) interferes with/disturbs the fucoidan-induced degradation of TGFRs and reduces fucoidan-mediated TGFRI/II ubiquitination in CL1-5 cells (A) Detection of Smurf2 and Smad7 binding on TGFR was assessed by the immunoprecipitation of TGFRI and TGFRII in CL1-5 cells treated with fucoidan (200 μg/ml) for 1 h. After incubation, whole cell lysates were immunoprecipitated overnight at 4°C using anti-TGFRI and anti-TGFRII antibodies. The protein levels of Smurf2, Smad7, and TGFRI/II were measured by western blotting assays, with the results showing the elevation of Smurf2 and Smad7 conjugation levels in TGFRs in the presence of fucoidan. (B) HEK293T cells were transfected with three types of sh-Smurf2 plasmid (sh-S1, sh-S2 and sh-S3) to produce lentiviruses as described in the Materials and Methods section. After harvesting the media, viruses containing individual Smurf2-shRNAs were infected into CL1-5 cells for 24 h, followed by puromycin selection; the expression of Smurf2 was detected by western blotting of the whole cell lysates. (C) CL1-5 cells (mock and knockdown, sh-Smurf2) were incubated with fucoidan (Fu, 200 μg/ml) for 3 h followed by western blotting of the whole cell lysates to detect the expression of TGFRI, TGFRII and Smurf2. Actin was used as an internal control, and the numbers below the actin row indicate the densitometric values normalized to the relative actin value. (D) Immunoprecipitation of TGFβ receptors (TGFRI and TGFRII) in CL1-5 cells was part of the *in vitro* ubiquitination assay. The two groups (mock and sh-Smurf2) of CL1-5 cells were pre-cultured in MG-132 (10 μM) for 30 min to inhibit proteasome activity and were subsequently treated with (Fu, 200 μg/ml) and without fucoidan (CTL) for 1 h. This experiment is representative of three similar experiments.

Smurf2 is one of the most important E3 ligases in the regulation of TGFR expression [30, 31]. Alternatively, Smad7, an adaptor protein, has a critical function in promoting Smurf2 conjugation on TGFRI, contributing to the process of TGFR hetero-complex degradation [31]. Here, we examined whether Smurf2 and/or Smad7 are involved in the processing step of the fucoidan-mediated UPP degradation of TGFR in lung cancer cells. As shown in Fig. 6A, during the fucoidan treatment of CL1-5 cells, there were significant increases in the amounts of Smurf2 and Smad7 proteins bound to TGFRI and TGFRII in the fucoidan-treated cells by 2-fold and 1-fold, respectively, compared with those in control cells. These results suggest that Smurf2 and Smad7 may be involved in fucoidan-enhanced, ubiquitin-dependent TGFR degradation.

Next, we performed Smurf2-knockdown experiments in CL1-5 cells. First, using Smurf2 shRNAs such as #3476 (sh-S1), #0792 (sh-S2), and #2881 (sh-S3), we found that Smurf2 knockdown leads to reductions in Smurf2 expression via western blotting analysis: 58%, 43%, and 47% reduction for sh-S1, sh-S2 and sh-S3, respectively (Fig. 6B). Second, we found that Smurf2 knockdown (sh-S2) substantially blocks the fucoidan-mediated reduction in TGFRI/RII expression in CL1-5 cells (Fig. 6C, samples 2 *vs* 4). Specifically, as expected, in the mock-transfected CL1-5 cells, fucoidan decreased TGFRI and TGFRII by 40% and 50%, respectively, compared with that in control cells (Fig. 6C, upper and lower panels, samples 1 *vs* 2). In contrast, in sh-Smurf2-transfected CL1-5 cells, no difference was noted in TGFRI expression with and without fucoidan treatment (Fig. 6C, upper panel, samples 3 *vs* 4), although a small effect of fucoidan was observed on TGFRII (Fig. 6C, lower panel, samples 3 *vs* 4). In contrast, both the TGFRI and TGFRII protein levels were drastically increased in sh-Smurf2-transfected CL1-5 cells but not in mock-transfected cells (Fig. 6C, upper and lower panels, samples 4 vs 2), consistent with the results of Smurf2 knockdown leading to the reduction in Smurf2 expression (Fig. 6B).

### Effect of Smurf2 knockdown on the fucoidan-mediated UPP in TGFRI/II protein degradation in CL1-5 cells

Moreover, to further explore the role of Smurf2 in the fucoidan-induced degradation of TGFR proteins, an *in vitro* ubiquitination activity assay was conducted against ubiquitin proteins to examine the role and involvement of Smurf2 in the fucoidan enhancement of UPP-mediated TGFRI/II protein degradation in sh-Smurf2-transfected CL1-5 cells. The cells were pre-incubated with MG-132 and treated with fucoidan, and then the whole cell lysates were incubated with anti-TGFRs antibodies. The immunoprecipitated proteins were next assessed using an anti-ubiquitin antibody. Regarding TGFRI, in mock- and sh-Smurf2-transfected cells, a higher level of TGFRI ubiquitination was found in cells treated with MG-132 and fucoidan than in cells treated with MG-132 alone (Fig. 6D-I, samples 2 and 4 *vs* 1 and 3, respectively). In essence, the incubation of sh-Smurf2-transfected cells with MG-132, followed by fucoidan treatment, showed a higher level of TGFRI ubiquitination—i.e., the intensity of the smeared bands of degenerated TGFRI protein in the fucoidan-treated cells—than that in control cells (Fig. 6D-I, samples 4 *vs* 3). These results suggest that even when sh-Smurf2 knocks down endogenous Smurf2, fucoidan may detour through a “Smurf2-dependent pathway” to enhance the degradation of TGFRI; however, we could not eliminate the possibility that the Smurf2-shRNAs we used were less efficient in the knockdown of the endogenous Smurf2 functions in the tested cells.

Considering the role of Smurf2 in the fucoidan-induced decrease in TGFRII proteins via UPP, we found a higher level of fucoidan-induced TGFRII ubiquitination (i.e., a stronger intensity of the smeared bands of degenerated TGFRII protein) in the mock-transfected CL1-5 cells than in the sh-Smurf2-transfected cells (Fig. 6D-II, samples 2 *vs* 4), as expected. We also found dissimilar patterns for TGFRII and TGFRI proteins in sh-Smurf2-transfected cells. Briefly, less of a difference was noted in TGFRII ubiquitination in sh-Smurf2-transfected cells treated with or without fucoidan (Fig. 6D-II, samples 4 *vs* 3).

Taken together, our results indicate that the smeared bands consisted of polyubiquitinated TGFRI/II protein, and we are the first to demonstrate that the UPP is involved in fucoidan-mediated and fucoidan-enhanced TGFR degradation in lung cancer cells. In addition, we found that Smurf2 plays an important role in the assembly of the UPP in TGFR degradation.

### Fucoidan inhibits TGFβ-dependent mobility (migration and invasion) and down-regulates FAK signaling pathways in lung cancer cells

**Figure 7:**
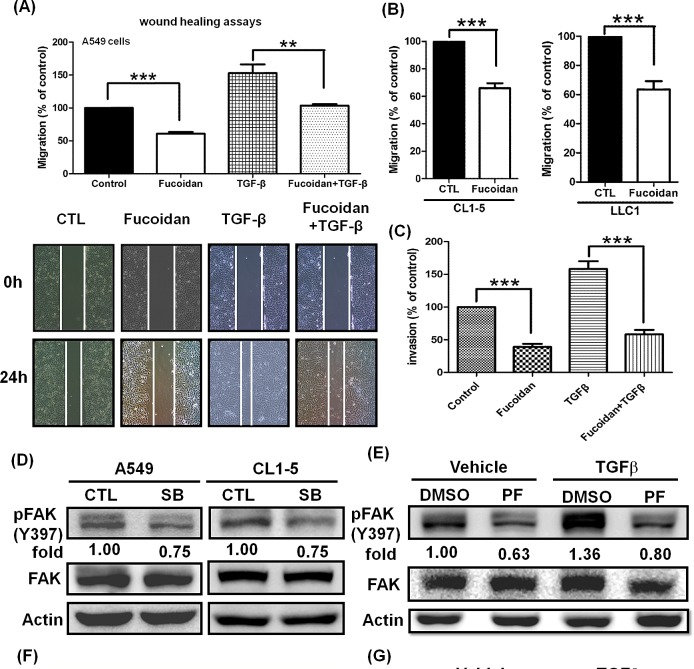
Fucoidan inhibits the phosphorylation of FAK and suppresses the TGFβ-dependent migratory and invasive properties of lung cancer cells (A) Wound closure assays for the effect of fucoidan on A549 cells. Images were captured at 0 and 24 h in serum-free medium with TGFβ (10 ng/ml) or without (control) in the presence of vehicle (PBS) or fucoidan (200 μg/ml). Total magnification: × 40. (B) To assess the migration potential, the highly metastatic CL1-5 and LLC1 cells were treated with fucoidan (200 μg/ml) for 24 h and then subjected to the Transwell^®^ assay. (C) A549 cell invasion was assayed in the presence of fucoidan alone (200 μg/ml) or fucoidan in combination with TGFβ (10 ng/ml). A549 cells (5×10^5^ cells/well) were seeded into the upper chamber with DMEM, with the bottom chamber coated with Matrigel^®^ and the lower chamber containing DMEM with 10% fetal bovine serum (FBS). The cell migration and invasion assays were performed for 24 h in a humidified incubator at 37°C with 5% CO_2._ Migratory and invading cells were counted under the microscope after fixation and staining with crystal violet. The data are expressed as the percentage of migration or invasion through the membrane or Matrigel^®^ matrix. The numbers of cells that migrated were observed and counted using an Olympus IX70 Inverted Microscope and SPOT Advanced Digital Imaging. One representative experiment of three independent experiments is shown. (D) The TGFR inhibitor SB431542 (SB) down-regulates the phosphorylation of FAK. A549 and CL1-5 cells were cultured in serum-free media for 24 h, followed by treatment with vehicle (-) or SB (20 μM) for 3 h and western blot analysis. (E) The FAK inhibitor PF573228 (PF, 2 μM) inhibits the TGFβ-induced phosphorylation of FAK. (F) PF573228 abolishes the TGFβ (10 ng/ml)-induced migration for 24 h in A549 cells. (G) Fucoidan suppresses the TGFβ-induced phosphorylation of FAK. A549 cells were cultured in serum-free media for 24 h and then treated with vehicle (-) or PF (2 μM) in (E) or with fucoidan (Fu, 200 μg/ml) in (G) for 30 min. The cells were then incubated with TGFβ (10 ng/ml) for 3 h and then subjected to western blot analysis. The proteins of whole cell lysates were immunoblotted with anti-pFAK (pY-397). FAK and actin served as internal controls for loading. One of three individual experiments is presented. Significant differences are shown (*P<0.05, **P<0.01 and ***P<0.005, compared with the control group).

In NSCLC, the TGFβ/TGFR pathway is considered as a tumor promoter, and the increasing TGFβ1 expression by tumor cells correlated with tumor progression and metastasis in lung cancer [33]. The migratory and invasive properties of metastasis are hallmarks of malignancy. Here, we examined and characterized the role of fucoidan in the migration and invasion of lung cancer cells. To maximize the inhibitory effects of fucoidan on cell migration and invasion activity in NSCLC (e.g., A549 and CL1-5 cells), the cells were stimulated with TGFβ, which has been shown to activate TGFRs relevant to oncogenic signaling in tumor mobility. Initially, wound healing assays were performed to determine TGFβ-induced migration *in vitro*; we found that fucoidan inhibits the migration of A549 with or without TGFβ by more than 40% and 50%, respectively, compared with that of control cells (Fig. 7A). In addition, using the Transwell^®^ assay, we found that fucoidan inhibits the migration of the highly metastatic CL1-5 and LLC1 cells (Fig. 7B). Moreover, using an invasion chamber—i.e., the Transwell^®^ assay in which cells invade across an extracellular matrix— we further elucidated and examined the inhibitory effect of fucoidan on the invasion of A549 cells with or without exogenous TGFβ. As shown in Fig. 7C, we found that fucoidan significantly inhibits the invasive properties of A549 cells with or without TGFβ by more than 70% and 60%, respectively, compared with those of control cells. Similar results were found in an invasion assay using CL1-5 cells (data not shown). In contrast, we found that fucoidan enhances the expression of E-cadherin but reduces the expression of N-cadherin and Vimentin at 24 and 48 h in CL1-5 cells in a dose-dependent manner (data not shown), suggesting that fucoidan inhibits cell mobility and alters the EMT in lung cancer cells.

Our results indicated that fucoidan promotes TGFR degradation and inhibits cell mobility. Focal adhesion kinase (FAK) is known to be involved in migration and invasion as well as metastasis. We were interested in examining the effect of fucoidan on FAK in the presence of TGFβ1. Initially, we investigated whether TGFR involves in the regulation of FAK in NSCLCs. Using a TGFR kinase inhibitor, SB431542 (SB), we found that SB down-regulates the phosphorylation of FAK by approximately 25% in A549 and CL1-5 cells (Fig. 7D), indicating that TGFR kinase activity may be related to the phosphorylation of FAK in lung cancer cells. Next, in the presence of an FAK inhibitor, PF573228 (PF), we found that PF decreases the TGF1-induced FAK phosphorylation (Fig. 7E) and migration (Fig. 7F) in A549 cells; similar results were found in CL1-5 cells (data not shown). The expression and activity of FAK have been reported to be up-regulated in lung cancer cells and to correlate with both malignant and metastatic disease [34]. Here, we also examined the effect of fucoidan on the phosphorylation of FAK. We found that fucoidan inhibits the phosphorylation of FAK in a dose-dependent manner (data not shown). Thus, to further investigate the effect of fucoidan on the TGFβ1 induction of FAK phosphorylation, we found that fucoidan abolishes approximately 40% of the TGFβ1-induced phosphorylation of FAK (Fig. 7G). Taken together, these results indicated that fucoidan not only induces TGFR degradation but also partially down-regulates FAK signaling pathways, suggesting that fucoidan suppresses the tumorigenesis of lung cancer via multiple pathways.

## DISCUSSION

TGFβ1, a multifunctional cytokine, is aberrantly over-expressed in various types of cancer cells and is involved in cell proliferation, migration, invasion, and metastasis. Importantly, many studies have indicated that TGFβ induces tumor progression and metastasis during the late stages of lung cancer carcinogenesis [13, 15, 32]. However, fucoidan also exerts multiple biological functions, including anti-cancer and immunoregulatory functions. We focused on examining the effects of fucoidan on TGFβ/TGFβR-mediated signaling pathways relevant to anti-lung cancer. We found that fucoidan inhibits tumorigenesis in LLC1 cell-xenograft mice and reduces the proliferation and mobility of NSCLC. Interestingly, in the “control-start-stop-continuous” experiments (Fig. 2), we further demonstrated much smaller tumor volumes/weights in LLC1-bearing mice continuously orally fed with fucoidan compared with mice fed with ddH_2_O and mice discontinuously fed with fucoidan. Surprisingly, the tumor volumes/weights of LLC1-bearing mice that began oral feeding with fucoidan on day 13 (EXP 1) were nevertheless smaller than those of the control group. This reduction in tumor volume/weight suggests that fucoidan exerts both preventive and therapeutic functions in lung cancer. In addition, continuous treatment with fucoidan has greater “efficacy” in reducing the tumor volume and inhibits the expression of TGFRI and TGFRII. We also found that AST expression is significantly lower in LLC1-xenograft mice with continuous fucoidan oral feeding (fucoidan) than in the control group of mice (CTL). Because the presence of AST is used to detect liver damage as a clinical biomarker of liver injury in patients/animals with some degree of intact liver function, our current findings indicate that fucoidan exerts an influence on neither liver function nor liver toxicity in mice. Moreover, our results suggest that fucoidan not only inhibits tumorigenesis but also plays a protective role regarding the health of liver organs. In addition, one of our main objectives in studying the effect of fucoidan on NSCLCs and xenograft mice is to prepare for the use of fucoidan as a nutritional/dietary supplement, rather than as a drug-like therapy, for cancer patients in the future. Therefore, we do not investigate the pharmacokinetics of oral fucoidan in the current study.

Upon ligand binding, TGFRII recruits and phosphorylates TGFRI, consequently triggering the activation of multiple complicated down-stream signaling cascades. The TGFβ/TGFR signal transduction pathways are regulated at multiple levels by ubiquitin-mediated targeting [28] and proteasomal degradation [35]. Notably, we first found that fucoidan leads to a reduction in the protein levels of TGFRI and TGFRII in lung cancer cells *in vitro* and *in vivo*, in agreement with our previous reports that fucoidan decreases metastasis by enhancing the UPP in TGFR degradation in breast cancer [4]. Furthermore, Smurf2 has been reported to be one of the most important E3 ligases that regulate TGFR degradation [36]. Here, we are the first to demonstrate that fucoidan enhances the amount of Smurf2/Smad7 conjugating with (binding to) TGFRI in NSCLC. Above all, fucoidan induction of the ubiquitin proteasome-mediated pathway in TGFR degradation is correlated with Smurf2/Smad7 activity, indicating that fucoidan either induces the activity of Smurf2 or triggers other proteins or pathways to cooperate with Smurf2 in the regulation of TGFR protein expression.

Considering the effect of fucoidan on NSCLCs in our studies, some questions may be raised, such as the types of responses (extracellular or intracellular) that occur in fucoidan-treated cells, how fucoidan interacts with the cells, and whether fucoidan induces any signal transduction. Based on our previous studies on the effects of fucoidan on macrophages, we found that the binding of fucoidan to macrophage scavenger receptor-A (MSR-A) in human THP-1 macrophages triggered the tyrosine phosphorylation of many proteins, including phospholipase C-γ1 and phosphatidylinositol-3-OH kinase [37]. Moreover, in examining MSR-A-mediated signal transduction upon fucoidan stimulation, we found that fucoidan induces the cytokines tumor necrosis factor-α and interleukin 1-β in murine macrophages J774A.1 and human THP-1 macrophages [38]. In the current study, we detected MSR-A expression on the cell membrane of lung cancer cells including A549 and LLC1 cells (data not shown), which was also recently reported [39, 40]. We also found that fucoidan induced the secretion of certain cytokines by A549 and LLC1 cells (unpublished data). Thus, it is clear that fucoidan stimulates multiple signal transduction pathways in cancer cells; in the future, we will study the effects of fucoidan on specific signaling pathways in the tumorigenesis of cancer cells.

Interestingly, we found that fucoidan rapidly decreases TGFβ-mediated FAK phosphorylation. FAK has been reported to play an essential role in mediating the interaction between integrin and TGFRII and to facilitate the oncogenic conversion of TGFβ in mammary tumor progression and metastasis, although the specific mechanisms remain unknown in NSCLC [41]. In the present study, we also found that fucoidan and a TGFR kinase inhibitor could inhibit the auto-phosphorylation of FAK, suggesting that the non-Smad pathway of TGFR may involve FAK regulation; however, we could not eliminate the possibility that fucoidan-decreased TGFRs may also directly or indirectly involve integrin-mediated reduction of FAK signaling via the novel interaction and crosstalk process among integrin, TGFRI and RII [42]. In the future, the effects of fucoidan on the interaction and crosstalk among membrane receptors (e.g., TGFRs, integrin, MSR-A) that are relevant to lung cancer tumorigenesis will be further investigated.

In summary, our current results demonstrate that orally administered fucoidan significantly reduces tumor volume/weight and TGFR protein levels in LLC1-xenograft mice *in vivo*. We propose that one of the mechanisms for fucoidan-mediated inhibition of lung cancer *in vivo* and *in vitro* depends on promoting the ubiquitination and degradation of TGFRs via Smurf2/Smad7. Moreover, in our un-published data, we also found that fucoidan significantly inhibits LLC1 xenograft growth and down-regulates TGFRs when administered via intraperitoneal (IP) injection. Therefore, our results suggest that fucoidan (oral or IP) inhibits lung tumorigenesis in a mouse model. To dissect the mechanisms involved in the fucoidan reduction of tumor growth in LLC1-bearing mice, we further demonstrated that fucoidan suppresses cell proliferation *in vitro*, a finding that is relevant to tumorigenesis in lung cancer cells. Conversely, in TGFR functions relevant to tumorigenesis, fucoidan enhances the conjugation of Smurf2 and Smad7 with TGFRs as well as the Smurf2-dependent ubiquitin proteasome pathway (UPP)-mediated degradation of TGFR. Thus, our current findings indicate that fucoidan has great potential as a therapeutic intervention in controlling lung cancer and that the manipulation of the Smurf2-dependent UPP-mediated degradation of TGFR proteins may be an effective strategy for cancer patients.

## MATERIALS AND METHODS

### Cell cultures

Human NSCLC CL1-5 cells were obtained from Dr. P.-C. Yang (NTU, Taiwan). Human NSCLC A549 cells and mouse lung cancer LLC1 cells were purchased from the Bioresource Collection and Research Center (BCRC, Hsinchu, Taiwan). CL1-5 cells were cultured in Roswell Park Memorial Institute medium 1640 (RPMI medium 1640, GIBCO-Life Technologies), supplemented with 10% heat-inactivated fetal bovine serum (FBS, GIBCO-Life Technologies) and 2.0 g/L of NaHCO_3_. A549 or LLC1 cells were cultured in Dulbecco's modified Eagle's medium (DMEM, GIBCO-Life Technologies), supplemented with 10% FBS and 3.7 or 1.5 g/L of NaHCO_3_. Adherent cells were detached by incubation with trypsin-EDTA (Invitrogen Co., Carlsbad, CA). The cells were maintained in an incubator in an atmosphere of 5% CO_2_ at 37°C.

### Reagents and antibodies

The fucoidan powder sample was a gift (Hi-Q oligo-fucoidan^®^) from Hi-Q Marine Biotech International, Ltd. (Taiwan), and fucoidan of *Fucus vesiculosus* was purchased from Sigma-Aldrich Co. (St. Louis, MO). Fucoidan was dissolved in double-distilled H_2_O (ddH_2_O) and stirred at 25°C for 30 min. The dissolved solution was filtered using 0.22-μm sterile filters (Millipore®). The proteasome inhibitor MG-132 was purchased from CalBiochem, EMD Millipore (Billerica, MA). Recombinant human TGFβ1 was purchased from R&D Systems, Inc. (Minneapolis, MN). PD98059 was purchased from Cell Signaling (Beverly, MA). LY294002 was purchased from A.G. Scientific (San Diego, CA). Antibodies against p-Akt (S473), Akt, p-Smad2/3, Smad2/3, Smad4, β-actin, TGFβR I, and TGFβR II, normal rabbit IgG, rabbit IgG-HRP, mouse IgG-HRP, rat IgG-HRP (for immunoblotting) and protein A/G plus-agarose were obtained from Santa Cruz Biotechnology (Santa Cruz, CA); TGFRI, ubiquitin, Smurf2, Smad7, p-FAK (Y397), and rabbit IgG-HRP were purchased from GeneTex (Hsinchu, Taiwan, or USA); and TGFRII was from Cell Signaling (Beverly, MA).

### Cell extracts and western blotting analysis

Lung cancer cells were grown on 6-cm tissue culture plates and treated with fucoidan for the indicated times. The cells were then washed once with 1 ml of ice-cold PBS containing 1% Na_3_VO_4_; the cells were lysed using 40 μl of lysis buffer (10 mM HEPES [pH 7.9], 10 mM KCl, 0.1 mM EDTA, 0.1 mM EGTA, and a protease inhibitor cocktail). The protein concentration of the supernatants was then measured using the Bradford protein assay (Bio-Rad, Hercules, CA) with BSA (Thermo Fisher Scientific, Rockwood, TN) as a standard. For western blotting analysis, total cell lysates, either from lung cancer cell culture or from tumors obtained from xenografts, were subjected to sodium dodecyl sulfate-polyacrylamide gel electrophoresis (SDS-PAGE) and western blotting as described previously [4]. The specific proteins tested were visualized using an enhanced chemiluminescence (ECL) western blotting system. The expression of actin was used as an internal control.

### Cell viability assay using the MTT assay

Cells (1×10^4^ cells per well) were seeded in triplicate on a 96-well plate and incubated overnight before treatment with fucoidan (25–300 μg/ml) for 24–72 h. After incubation, 3-(4,5-dimethylthiazole-2-yl)-2,5-diphenyl tetrazolium bromide (MTT) dye was added, and the mixture was incubated for 2 h. The detailed procedure has been described previously [4].

### Animals and fucoidan treatment

Male C57BL/6 strains of mice were obtained from the National Laboratory Animal Center of Taiwan. Mice were aged 6–8 weeks and kept under a standard protocol approved by the Institutional Animal Care of National Yang-Ming University. Each mouse was injected with LLC1 cells (2×10^5^ cells per mouse) subcutaneously into the dorsum. To examine the effects of fucoidan on tumor volume, mice were randomly distributed into two groups and orally fed with either ddH_2_O (control group) or fucoidan (experimental group). The treatment period was as indicated, and we recorded the body weight and tumor volume (*V*) (calculated using the formula: *V =* length × width × thickness in mm^3^) of each tested mouse three times each week. On the day prior to the end of treatment, aspartate aminotransferase (AST) and alanine aminotransferase (ALT) levels were analyzed via blood collection from the tail vein. At the end of the treatment, the liver, lung, spleen, and tumors of the mice were surgically collected. For analysis of the expression of specific proteins in the tumors, the tumors were forced through a fine wire mesh in cold PBS containing 1% Na_3_VO_4_. The pellets were re-suspended in RBC lysis buffer and washed with cold PBS containing 1% Na_3_VO_4_. After removing RBCs, the cells were lysed in lysis buffer and clarified by centrifugation at 12,000 ×g at 4°C for 15 min for further western blotting analysis.

### Immunoprecipitation of TGFβ receptors for the *in vitro* ubiquitination assay

To assess the ubiquitin regulatory ability of fucoidan in CL1-5, A549, and LLC1 cells, the cells were pre-cultured in MG-132 (10 μM) for 30 min to inhibit proteasome activity, followed by treatment with or without fucoidan (200 μg/ml) for 60 and 150 min. The cells were extracted with lysis buffer and then centrifuged at 13000×g for 10 min at 4°C. The cleared supernatants were prepared for immunoprecipitation. The cell lysates (400 μg of protein) were immunoprecipitated overnight at 4°C with various primary antibodies (1 μg); protein A/G was added to the immunoprecipitates followed by incubation at 4°C overnight. The beads were collected by centrifugation at 10,000 × g for 2 min and washed 4 times in PBS containing protease inhibitors and phosphatase inhibitors. The immunoprecipitation complex was subjected to SDS-PAGE, followed by immunoblotting with anti-ubiquitin antibody to detect polyubiquitinated TGFR proteins. The results of the *in vitro* ubiquitination assays were visualized using an enhanced chemiluminescence western blotting system. The detailed procedure has been described previously [4].

### Design and transfection of specific short hairpin RNA oligonucleotides (shRNAs) targeting Smurf2

Based on the sequence of the human Smurf2 gene (NM_022739.3), three specific shRNAs targeting Smurf2 were obtained from the National RNAi Core Facility Platform (Academia Sinica, Taipei, Taiwan). The targeting sequences for Smurf2 are 5'-GTGTGGATACTTGAGAATGAT-3' (Smurf2 shRNA#3476, shS1), 5'-GTGGACTGCAGTCGTTTATTT-3' (Smurf2 shRNA#0792, shS2) and 5'-GCTGGATTTCTCGGTTGTGTT-3' (Smurf2 shRNA#2881, shS3), which are also described and shown in Supplementary Table S1. To produce stable clones of Smurf2-knockdown cell lines, we used *in vitro* gene delivery of lentiviral vectors as previously described [43-45]. Briefly, HEK293T cells were cultured in a 10-cm dish for 24 h. After reaching an 80% density/well, using the manufacturer's protocol, we transfected the cells with individual plasmids using the Lipofectamine 2000 reagent as follows: TRC library plasmid, pLKO.1-shRNA (Smurf2); packaging plasmid, pCMV-ΔR8.91 (containing gag, pol, rev genes); and envelope plasmid, pMD.G (VSV-G expression plasmid). After 24 h, the media of the plates were changed by removing the transfection reagent and replaced with BSA-containing growth media. At 40 and 64 h post-transfection, the media containing lentiviruses were harvested. In addition, the targeted CL1-5 cells were infected with the viral suspension and selected with puromycin (pLKO.1-puro) for future study and examination.

### Migration (or wound healing assay) and invasion assays

The migration activity of lung cancer cells was assessed using the Costar Transwell^®^ procedure. Briefly, cells (1×10^4^/each well) were seeded in the upper chamber in diluted 0.5% FBS-DMEM and treated with fucoidan (100 μg/ml). The cell migration ability over 24 h was then assessed. For the lung cancer cell invasion assay, Transwell^®^ filters coated with an appropriate amount of Matrigel^®^ (Becton Dickinson Biosciences) were used. Cells (5×10^4^/each well) were seeded in the upper chamber wells and incubated for 24 h. Cells that migrated through the basement membrane filter were fixed with 70% methanol for 10 min and stained with Liu's stain. The lower face of the membrane was examined, and the migrated cells were counted under a microscope. The detailed procedure has been described previously [4].

### Statistical analysis

All of the data are expressed as the mean ± standard error. Significant differences between two groups were determined by *t* test analyses using Microsoft Excel. A *p* value of less than 0.05 was considered statistically significant (*P < 0.05, **P < 0.01 and ***P < 0.005).

### SUPPLEMENTARY MATERIAL TABLE AND FIGURES


