# Supplementary material for: Fucoidan inhibition of lung cancer in vivo and in vitro: role of the Smurf2-dependent ubiquitin proteasome pathway in TGFβ receptor degradation
Source: Oncotarget. 2014 Aug 6;5(17):7870–85. doi: 10.18632/oncotarget.2317 (PMC4202167; doi:10.18632/oncotarget.2317)
Supplement: Supplementary file 1 [file oncotarget-05-7870-s001.pdf]

Fucoidan inhibition of lung cancer *in vivo* and *in vitro*: role of the Smurf2-dependent ubiquitin proteasome pathway in TGFβ receptor degradation

Supplementary Material

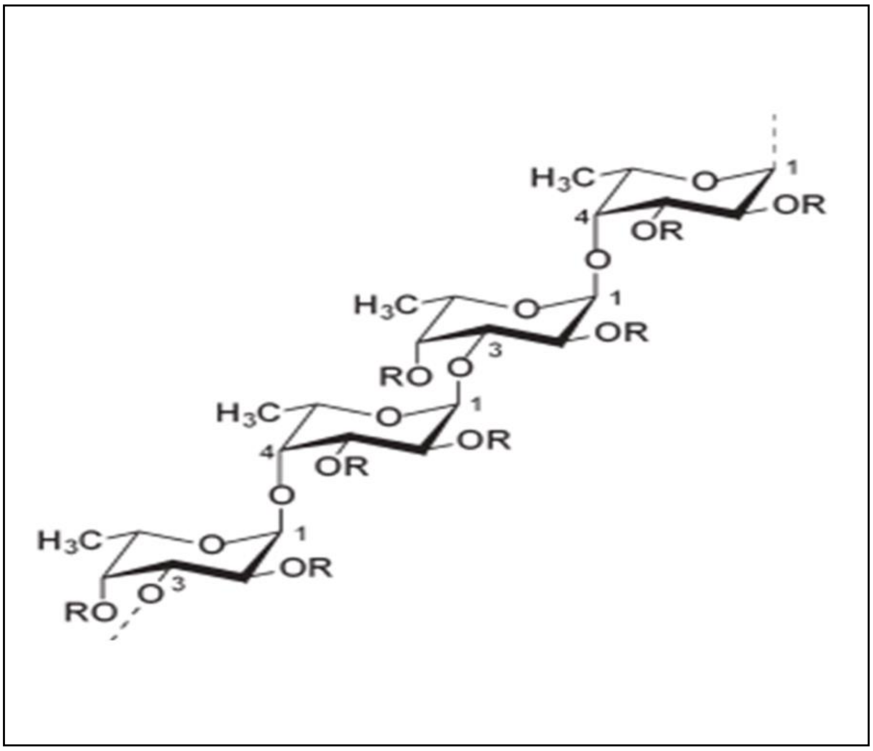

Scheme A. The proposed structure of fucoidan

Supplementary Table S1: Sequences of shRNA for Smurf2

shRNA	Target sequences (5' to 3')	shRNA sequences (5' to 3')
Smurf2 #3476 (sh-S1)	GTGTGGATAC-TTGAGAATGAT	CCGGGTGTGGATACTTGAGAATGATCTCGA-GATCATTCTCAAGTATCCACACTTTTT
Smurf2 #2881 (sh-S2)	GTGGACTGCA-GTCGTTTATTT	CCGGGTGGACTGCAGTCGTTTATTTCTCGA-GAAATAAACGACTGCAGTCCACTTTTTG
Smurf2 #0792 (sh-S3)	GCTGGATTTCT-CGGTTGTGTT	CCGGGCTGGATTTCTCGGTTGTGTTCTCGA-GAACACAACCGAGAAATCCAGCTTTTT

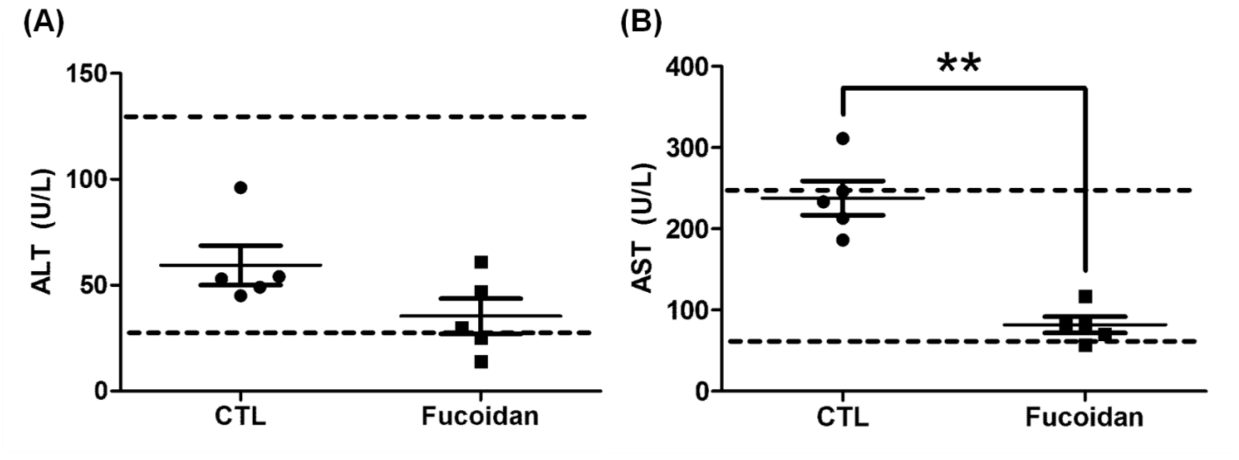

Fig. S1: The effects of fucoidan on expression of aspartate aminotransferase (AST) and alanine aminotransferase (ALT) relevant to liver functions of LLC1-xenograft male C57BL6 mice. The AST and ALT of mice sera from tail vain blood collection were analyzed. Statistically significant differences are shown compared to the control group (***P*<0.01). One representative of three independent experiments is shown; n = 5.

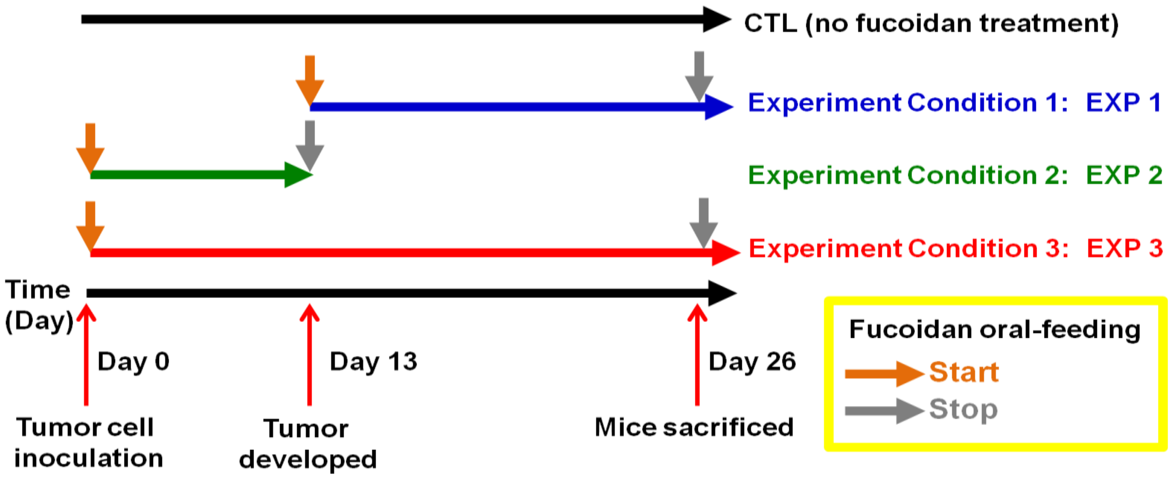

Fig. S2: The schematic design for the experiments of Fig. 2.

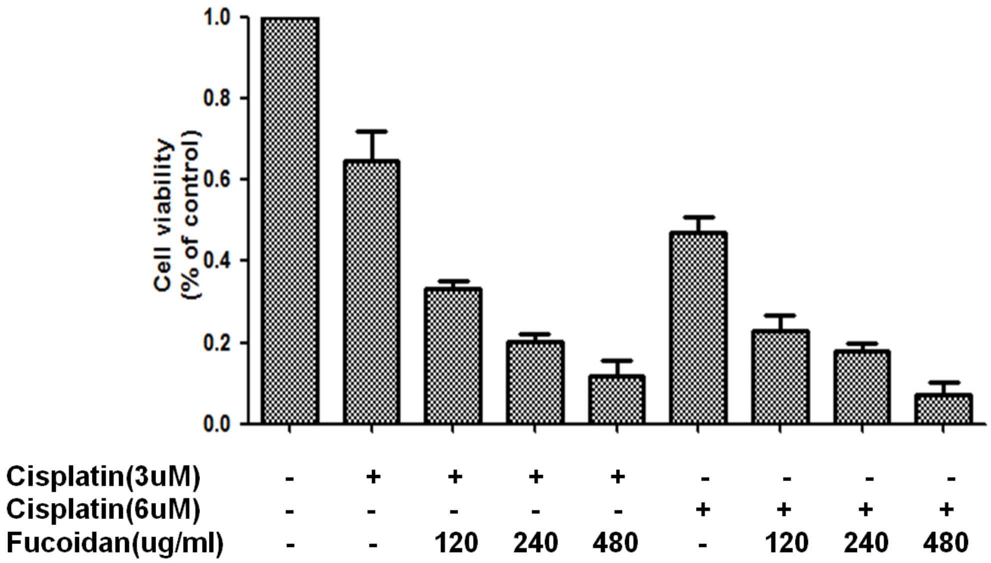

Fig. S3: The synergistic cytotoxic effects of fucoidan combined with cisplatin on the cell viability of LLC1 cells. LLC1 cells were co-treated with various dosages of fucoidan (0-480 μg/ml) and cisplatin (3 or 6 μM) for 24 h; the viability of cells was determined by MTT assay. Each group of fucoidan is normalized against each untreated control. The data are a representative of three separate experiments and are presented as the mean ± SD; error bars indicate SD.

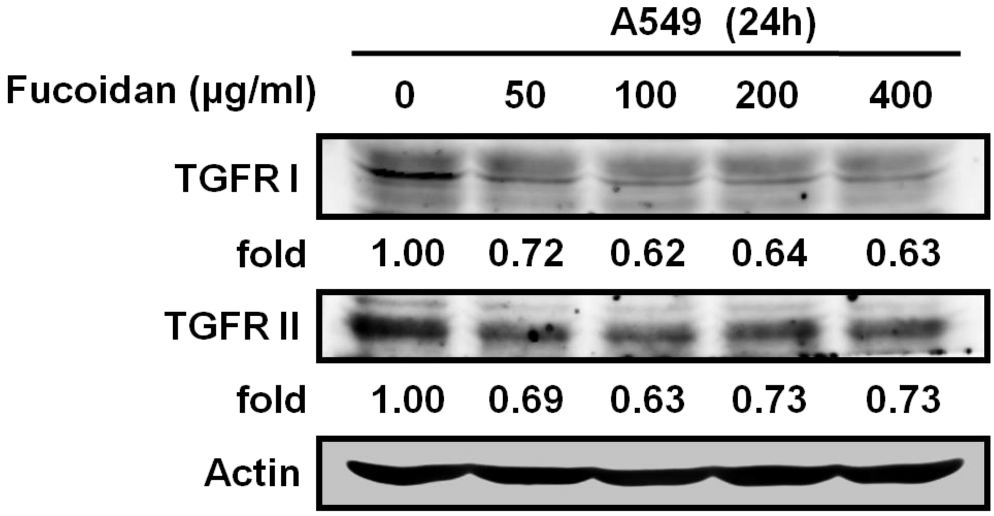

Fig. S4: Fucoidan downregulates TGFRI/II proteins levels in A549 cells at 24 h. A549 cells were treated with various dose of fucoidan (0-400 μg/ml) for 24 h. This was followed by western blotting assays of the whole cell lysates to detect the expression of TGFR I and TGFR II proteins. The actin was used as the internal control and the numbers (folds) below the TGFR I and TGFR II rows indicate the densitometric values normalized against the relative actin value.

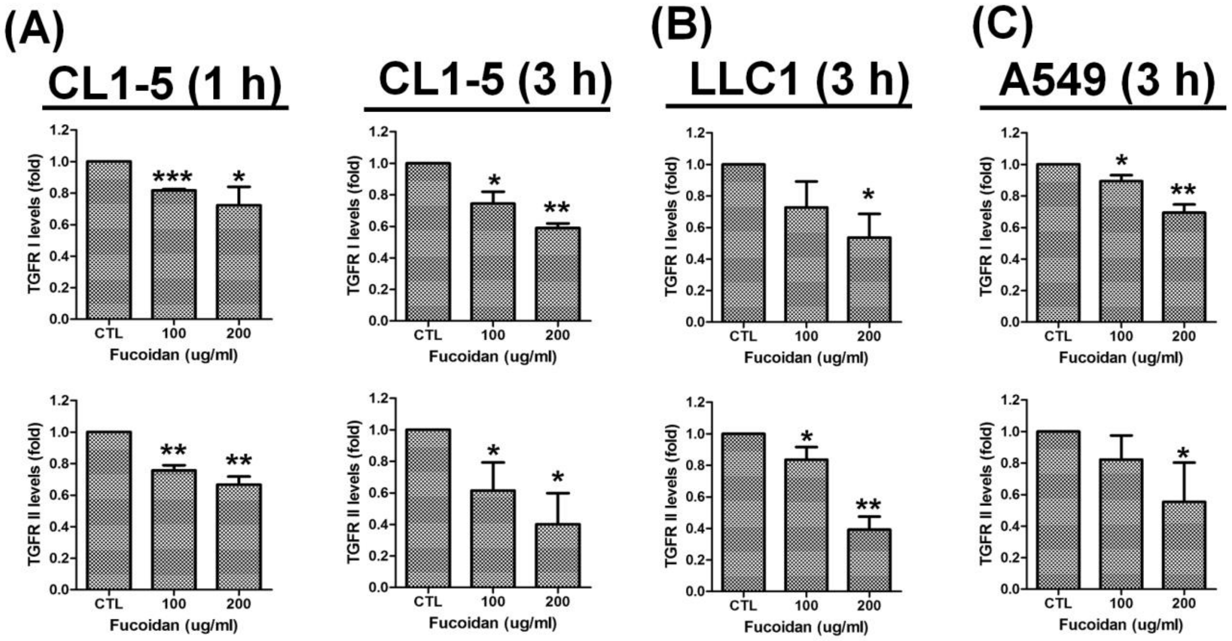

Fig. S5: Quantification of fucoidan downregulated TGFRI/II proteins levels in Figure 4.

Quantification of the intensities of the bands of TGFRI and TGFRII in the experiment is the representative of three separate determinations by ImageJ (National Institute of Mental Health, Bethesda, MD, USA). The data are presented as the mean ± SD; error bars indicate SD. Significant differences are shown (*$P < 0.05$, **$P < 0.01$ and ***$P < 0.001$, compared with the control group).
